# Supplementary material for: Structure of human DPPA3 bound to the UHRF1 PHD finger reveals its functional and structural differences from mouse DPPA3
Source: Commun Biol. 2024 Jun 19;7:746. doi: 10.1038/s42003-024-06434-9 (PMC11187062; doi:10.1038/s42003-024-06434-9)
Supplement: Supplementary file 2 — Supplementary information [file 42003_2024_6434_MOESM2_ESM.pdf]

Supplementary Information

Structure of human DPPA3 bound to the UHRF1 PHD finger reveals its functional and structural differences from mouse DPPA3

Nao Shiraishi, Tsuyoshi Konuma, Yoshie Chiba, Sayaka Hokazono, Nao Nakamura, Md Hadiul Islam, Makoto Nakanishi, Atsuya Nishiyama, Kyohei Arita

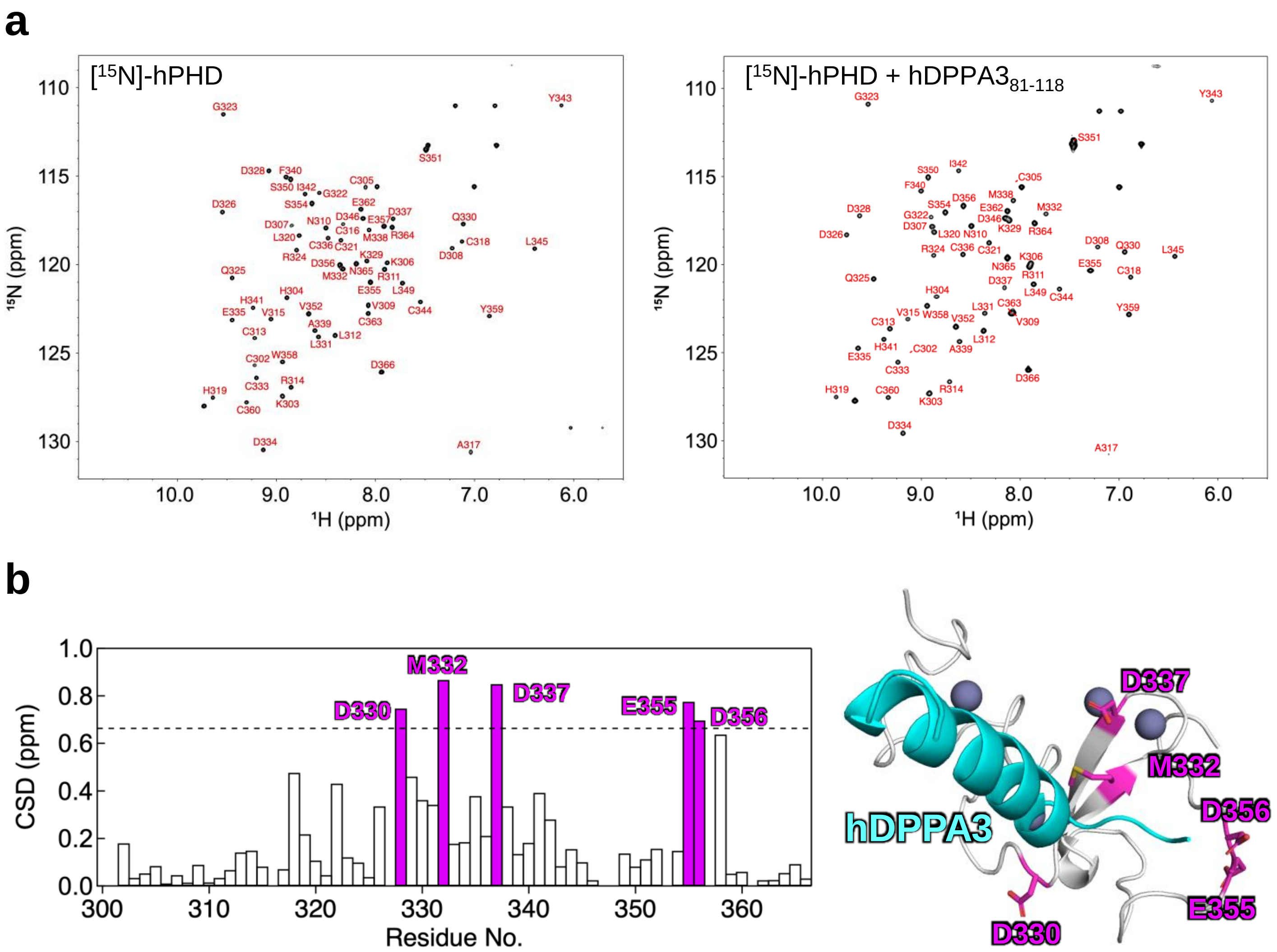

Supplementary Figure 1: Nuclear magnetic resonance (NMR) titration experiments

(a) Signal assignments in the ^{1}H-^{15}N HSQC spectra of hPHD in the free state (left) and complex state with hDPPA3$_{81-118}$ (right). **(b)** Weighted average chemical shift differences in ^{1}H and ^{15}N resonances between free hPHD and hPHD in complex with hDPPA3$_{81-118}$ (left). The dashed line represents the mean plus 2 standard deviations. Mapping of D330, M332, D337, E355, and D356 in the crystal structure (right).

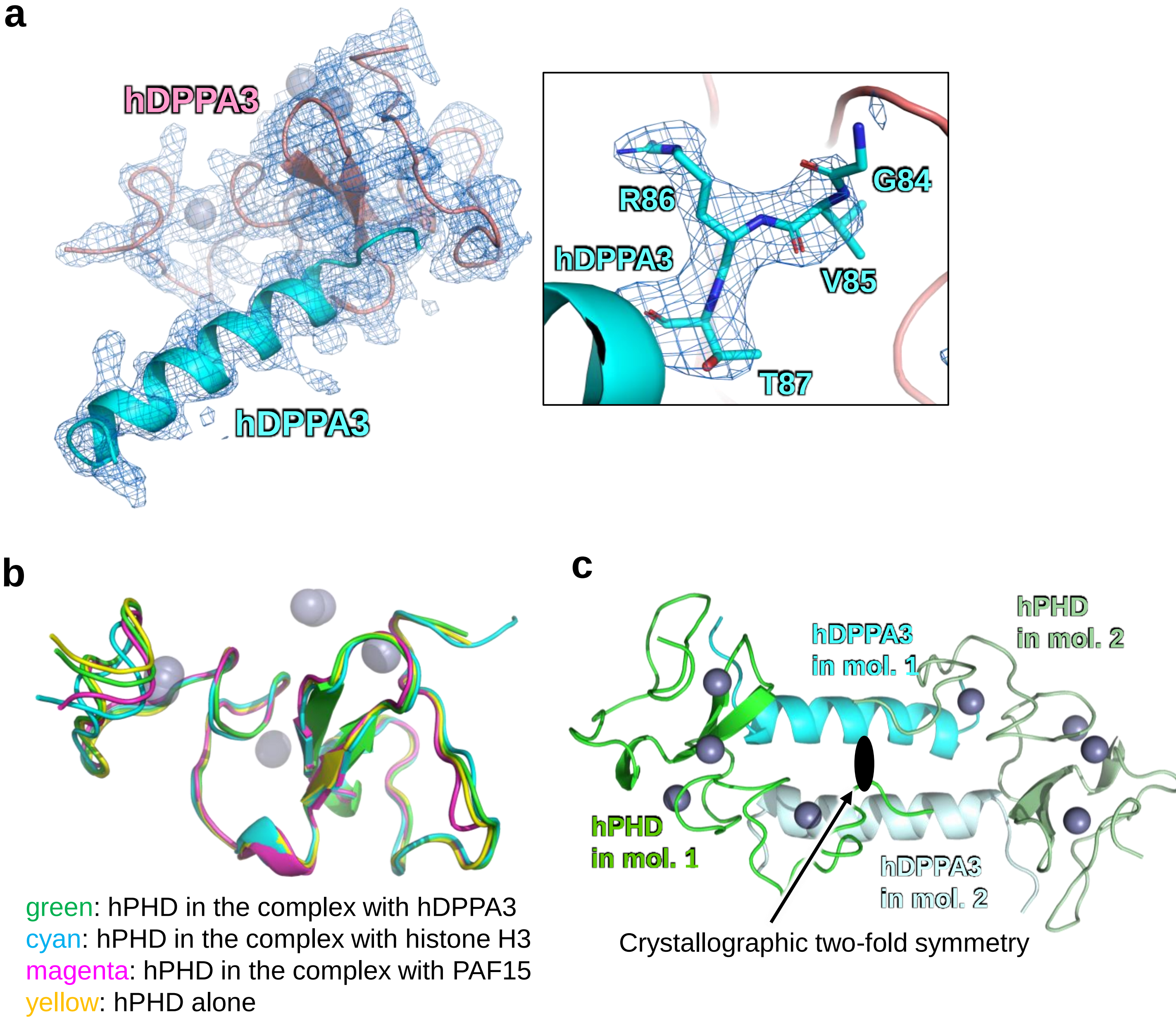

Supplementary Figure 2: Structural features of the hPHD:hDPPA3 complex in crystal form

(a) Overlay of 2|*F*o|-|*F*c| map countered at 1.0 σ (blue mesh) on the cartoon model of the hPHD:hDPPA3$_{81\text{-}118}$ complex. The figure on the right shows a close-up view of the VRT motif of hDPPA3. The Omit map contoured at 3 σ is shown as a blue mesh. **(b)** Structural comparison of the human UHRF1 PHD finger in the complex with hDPPA3 (green), histone H3 (cyan), and PAF15 (magenta) and of the structure of hPHD alone (yellow). **(c)** The hPHD:hDPPA3$_{81\text{-}118}$ complex forms a dimer with a crystallographic symmetry-related molecule via interaction with the α-helix in hDPPA3. The crystallographic two-fold symmetry symbol is indicated.

a

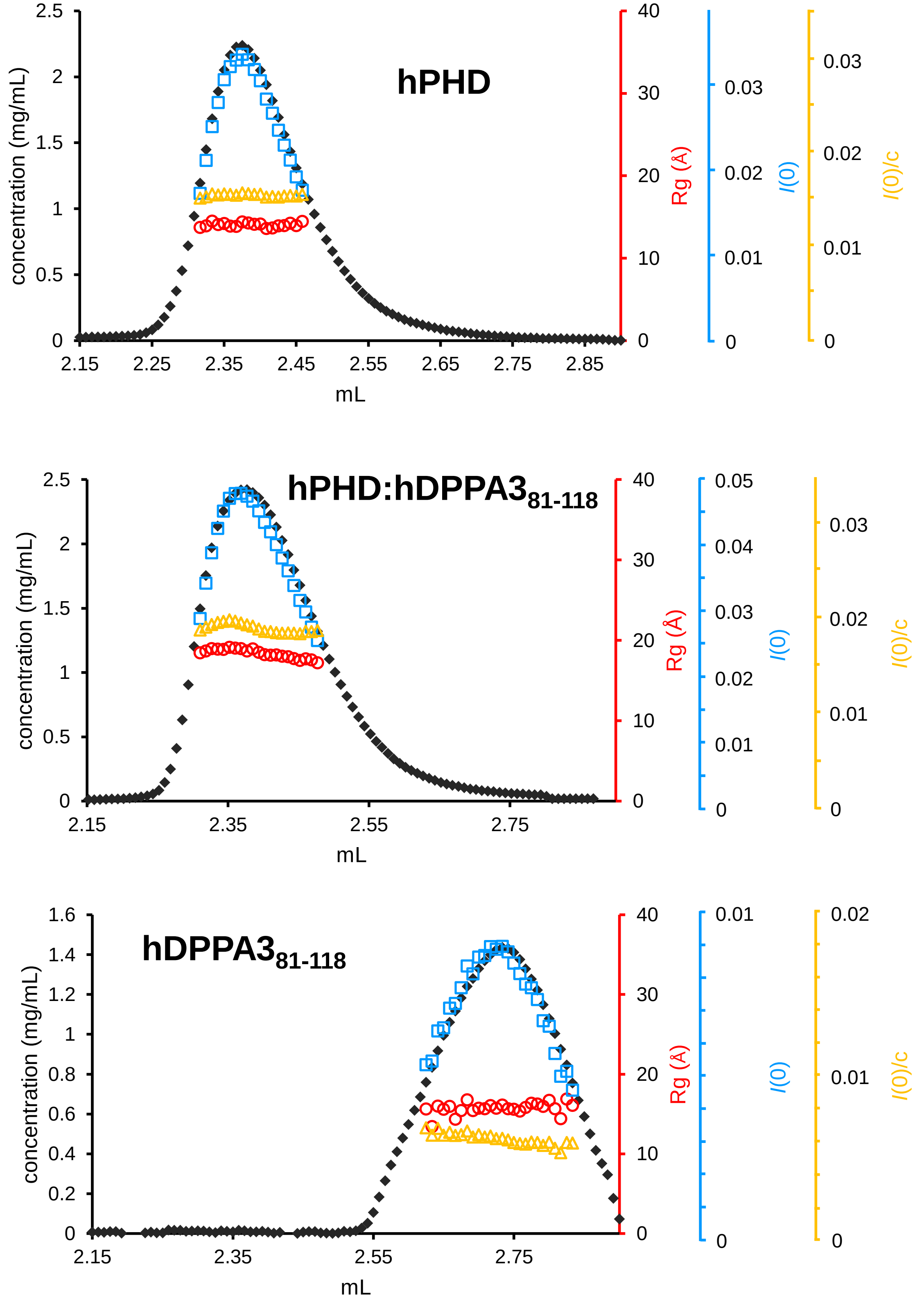
hPHD
concentration (mg/mL)
mL
Rg (Å)
I(0)
I(0)/c
hPHD:hDPPA3 81-118
concentration (mg/mL)
mL
Rg (Å)
I(0)
I(0)/c
hDPPA3 81-118
concentration (mg/mL)
mL
Rg (Å)
I(0)
I(0)/c

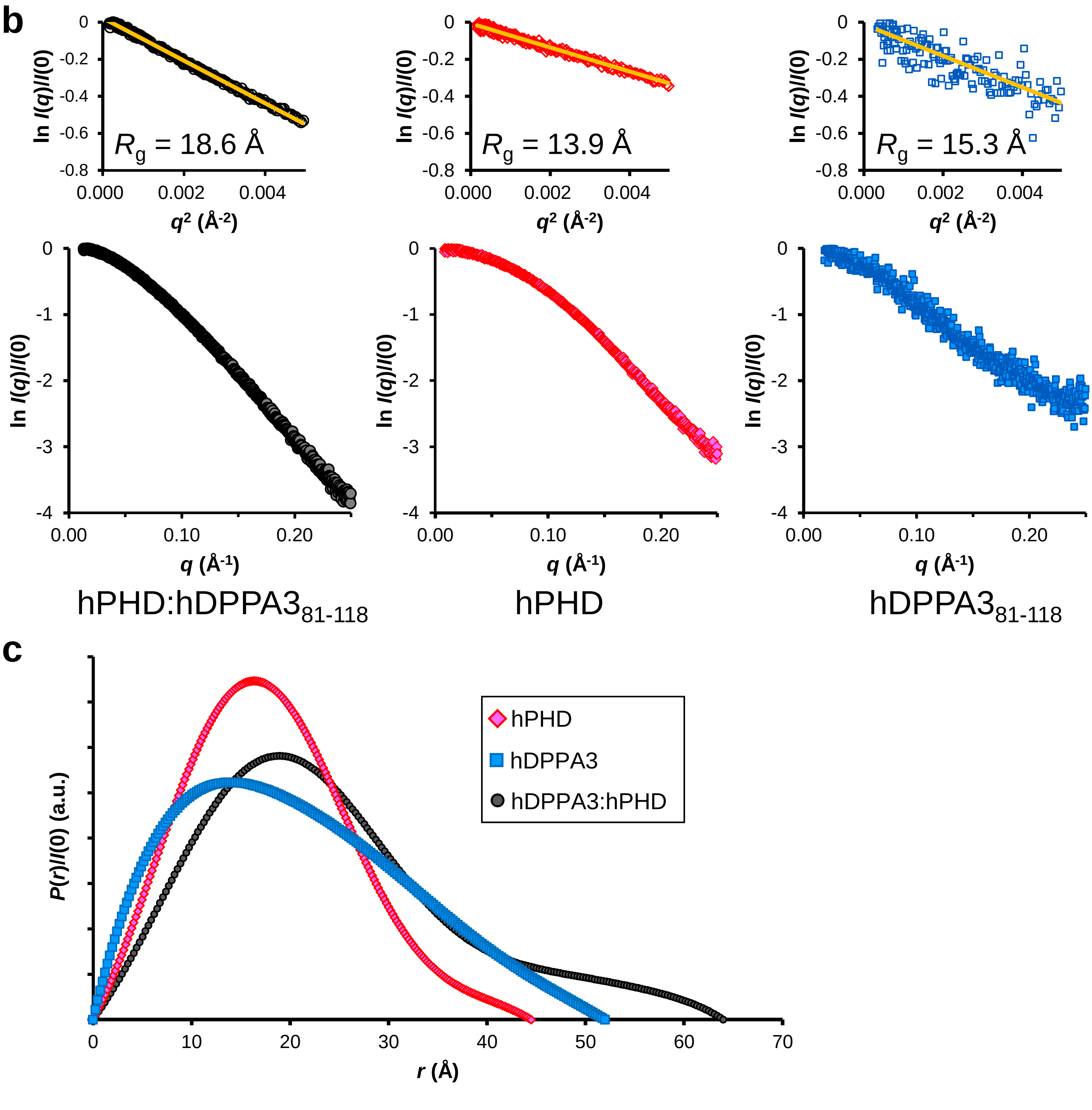

Supplementary Figure 3: Size-exclusion chromatography–small-angle X-ray scattering (SEC-SAXS) data.

(a) SEC-SAXS data of hPHD (top), hPHD:hDPPA3$_{81\text{-}118}$ complex (middle), and hDPPA3$_{81\text{-}118}$ (bottom). Absorption at 280 nm, *I*(0) calculated from the Guinier analysis, R_g values, and *I*(0)/*c* (concentration: mg/ml) are shown as black diamonds, cyan squares, red circles, and yellow triangles, respectively. **(b)** SAXS scattering intensity of the hPHD:hDPPA3$_{81\text{-}118}$ complex (left), hPHD (center), and hDPPA3$_{81\text{-}118}$ (right). The top panels indicate the Guinier plot with $qR_g < 1.3$ shown as an orange line. R_g values are shown in the graphs. **(c)** Overlay of the distance distribution function, P(r), of hPHD:hDPPA3$_{81\text{-}118}$ complex (black), hPHD (pink), and hDPPA3$_{81\text{-}118}$ (cyan).

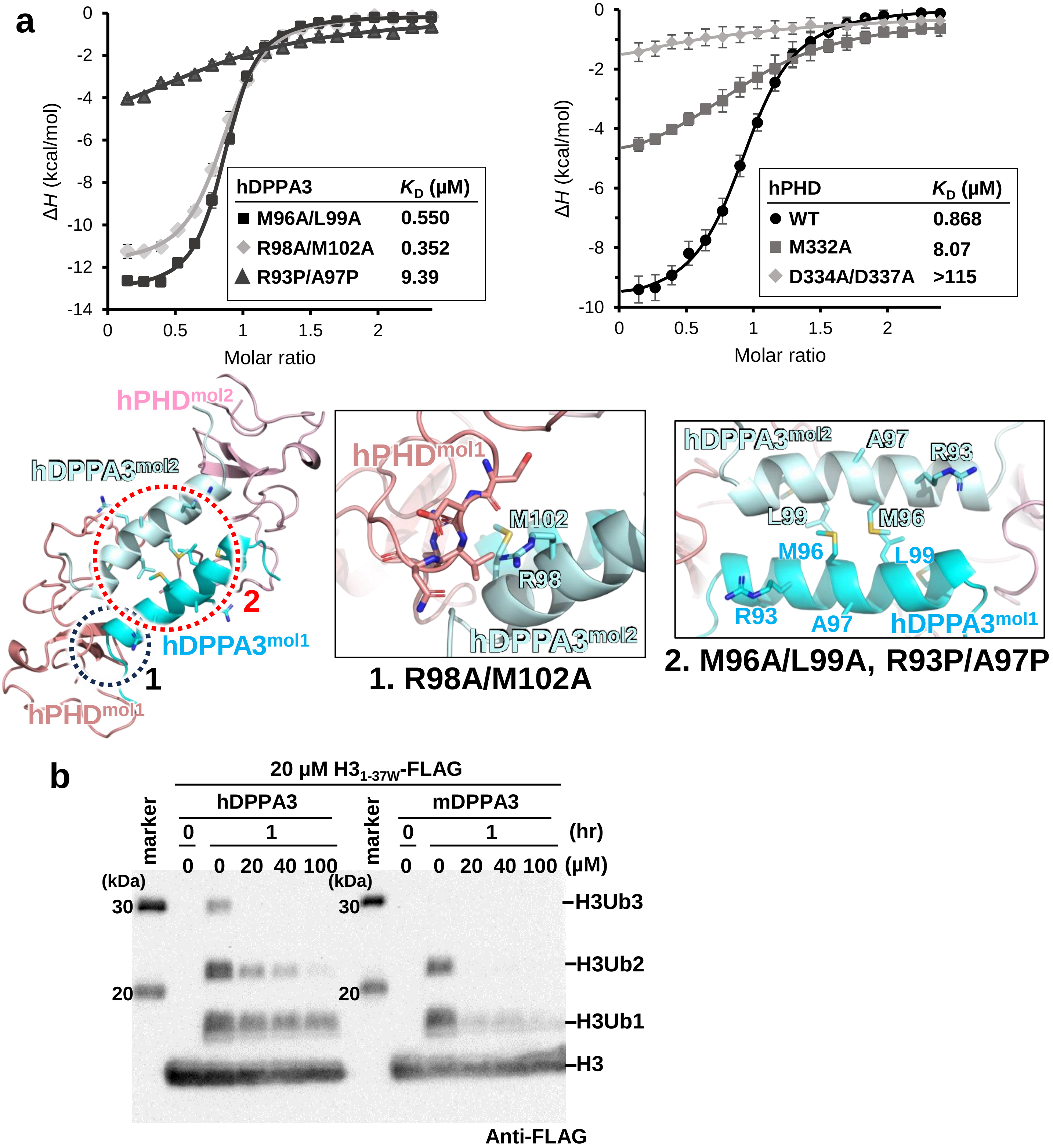

Supplementary Figure 4: Biochemical assays.

(a) Isothermal titration calorimetry (ITC) measurements for mutants of hDPPA3$_{81\text{-}118}$ and hPHD. Superimposition of enthalpy change plots with standard deviations. Data are presented as the mean ± SD for n = 3. The bottom figures represent the mutated residues (stick model) at the dimer interface in the crystal. **(b)** An *in vitro* ubiquitination assay to compare the inhibitory effects of hDPPA3 and mDPPA3. The gel image is representative of n = 3 independent experiments.

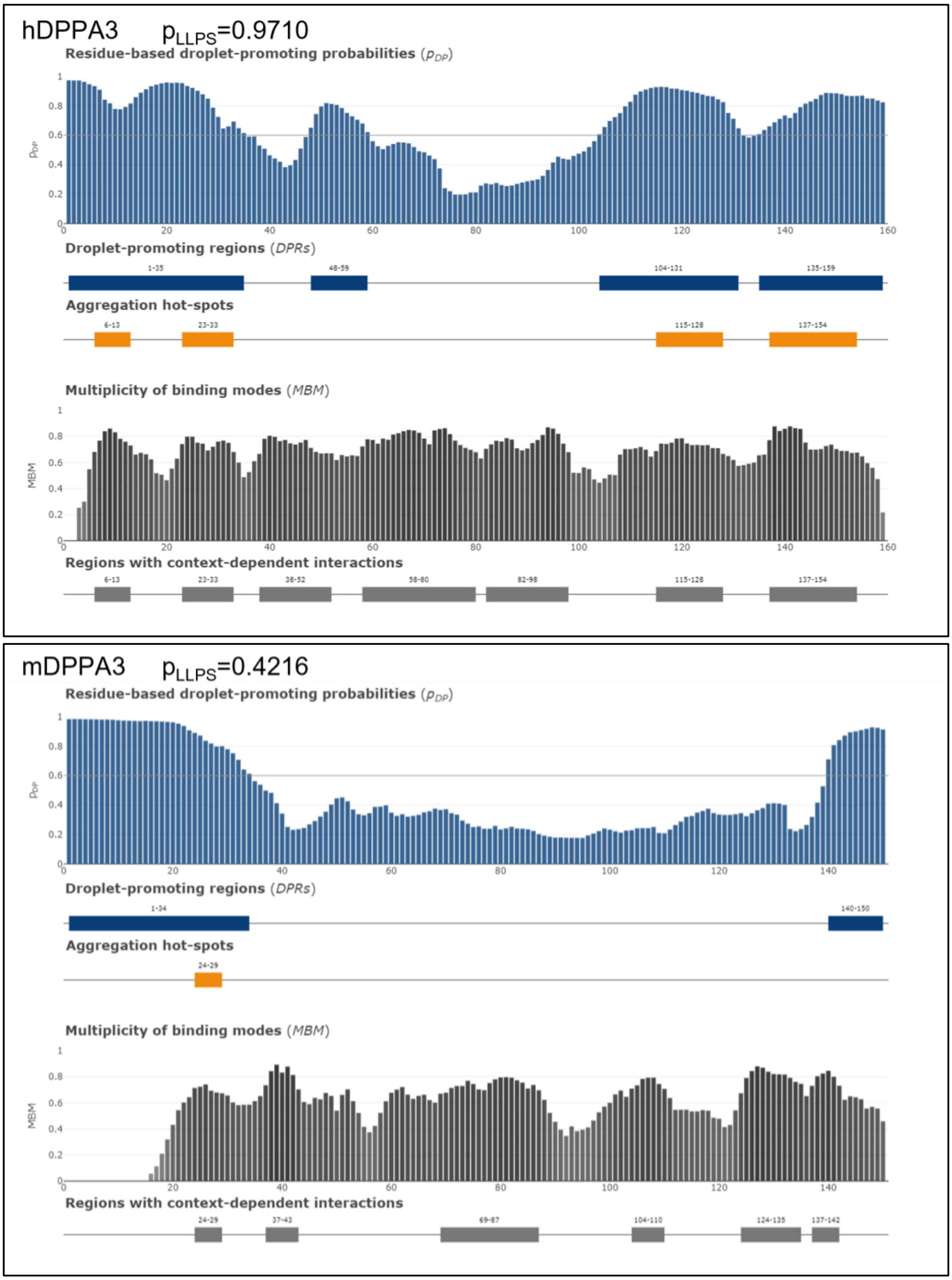

Supplementary Figure 5: Prediction of droplet formation in human (upper panel) and mouse DPPA3 (lower panel).

PLLPS is the probability of forming a droplet state through liquid-liquid phase separation. PLLPS ≥ 0.60 are assigned as droplet-drivers.

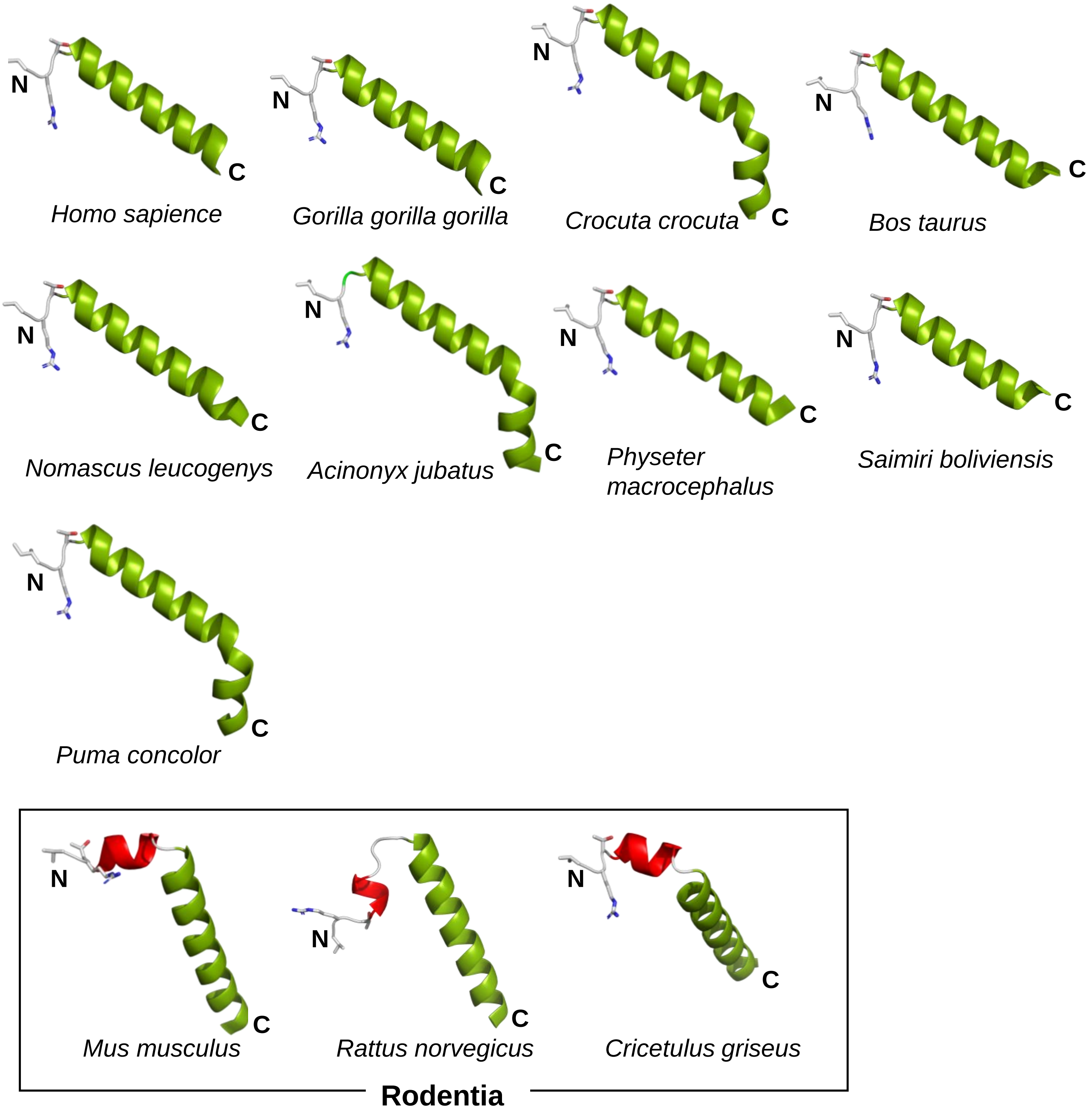

Supplementary Figure 6: AlphaFold 2 structural prediction of DPPA3 in various species.

The VRT motif and the following α-helix of DPPA3, predicted by AlphaFold2, are displayed. White sticks indicate side chains of the conserved VRT motif. α-Helices are shown as cartoon models, in which the long α-helix is colored green and the short α-helix, unique to Rodentia, is displayed in red.

a

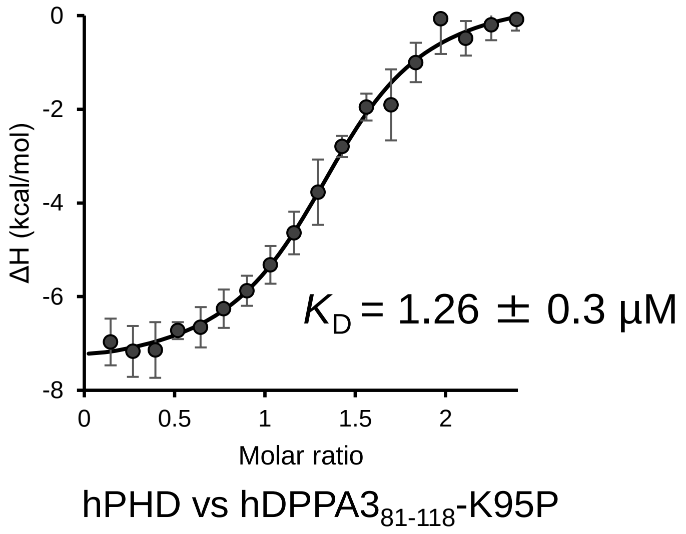

b

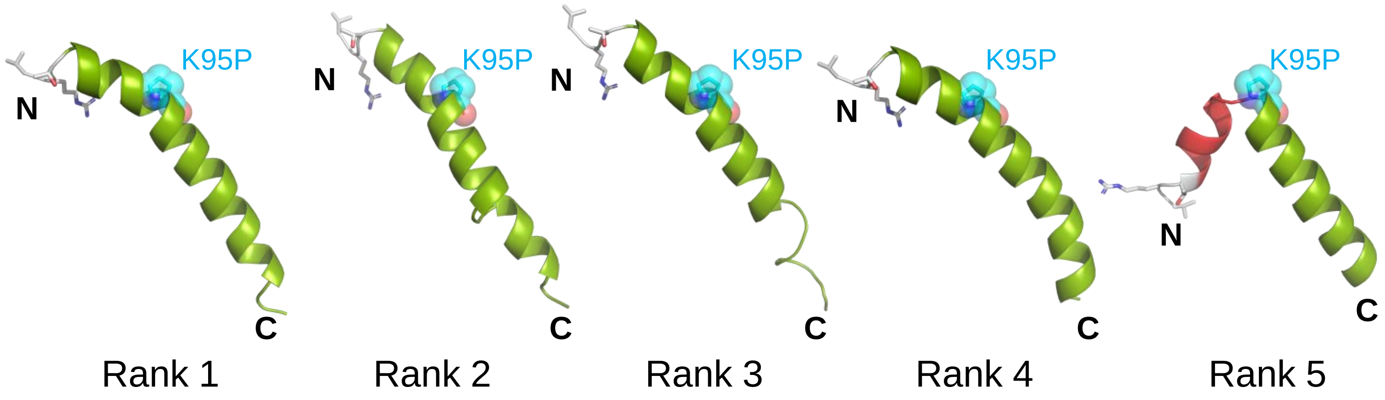

Supplementary Figure 7: Analysis of K95P mutant of hDPPA3.
(a) ITC measurements of the K95P mutant hDPPA3$_{81\text{-}118}$ and WT hPHD. Superimposition of enthalpy change plots with standard deviations. Data are presented as mean ± SD for n = 3.
(b) AlphaFold2 structure prediction of K95P mutant of hDPPA3$_{81\text{-}118}$. K95P is shown as a cyan stick superimposed on a transparent sphere. The VRT motif is depicted as a stick model.

Supplementary Table 1

Sample details

Sample name	**hPHD**	**hPHD:hDPPA3**	**hDPPA3**
Organism	*Homo sapiens sapiens*		
UniProt sequence ID	Q96T88 (UHRF1)	Q96T88 (UHRF1)<br>Q6W0C5 (DPPA3)	Q6W0C5 (DPPA3)
Extinction coefficient ε	8480	9970	1490
Calculated monomeric Mr from sequence (kDa	7.8 ($PHD_{299\text{-}366}$)	12.2:<br>[7.8 ($PHD_{299\text{-}366}$) + 4.4 ($DPPA3_{81\text{-}117}$)]	4.4 ($DPPA3_{81\text{-}117}$)
HPLC system	Nexera/Prominence-I (Shimazu)		
SEC Column	Superdex™ 200 Increase 5/150 GL		
Temperature (K)	293		
Injection volume (µL)	50		
Loading concentration (mg/mL)	12		
Flow rate (mL/min)	0.025		
SEC buffer	20 mM Tris-HCl (pH 7.5), 150 mM NaCl, 2 mM DTT, 10 µM Zinc acetate and 5% glycerol		

Data-collection parameters

Beamline	Photon Factory BL-10C		
Detector	Pilatus3 2 M		
Sample-to-detector distance (mm)	2,082		
Wavelength (Å)	1.5		
q range ($Å^{-1}$)	0.0083 - 0.264	0.00865 - 0.264	0.018-0.264
Exposure time (s)	20/frame		
Flux (photons/s)	$1.1{\times}10^{11}$		
Beam size	0.63 mm (H)×0.18 mm (V)		
Concentration range (mg/mL)	1.17 - 2.18	1.3 - 2.46	0.739-1.44
Absolute scaling method	Using the scattering intensity of water		
Normalization	To transmitted intensity by beam-stop counter		

Structural parameters

**Guinier analysis**			
I(0) (cm^{-1})	0.015 ± 1.8 E^{-5}	0.019 ± 2.2 E^{-5}	0.0057 ± 9.0 E^{-5}
R_g (Å)	13.9 ± 0.03 Å	18.6 ± 0.04 Å	15.3 ± 0.43 Å
q-range ($Å^{-1}$), point range	0.18-1.30, 14-248	0.20-1.30, 7-179	0.30-1.30, 3-186
***P*(r) analysis**			
I(0) (cm^{-1})	0.015 ± 8.6 E^{-5}	0.019 ± 1.2 E^{-4}	0.0058 ± 1.1 E^{-4}
R_g (Å)	14.0 ± 0.11 Å	19.1 ± 0.19 Å	16.5 ± 0.32 Å
D_{max} (Å)	44.5	64.0	52.0
q-range ($Å^{-1}$), point range	0.0128-0.2642, 14-742	0.0100-0.2156, 5-600	0.0211-0.2642, 10-714
Porod volume estimate ($Å^3$)	16393	16873	13315
Dry volume calculated from sequence ($Å^3$)	9381	14711	5352
Partical specific volume (cm^3g^{-1})	0.743	0.743	0.743
Molecular mass Mr [from V_c] (kDa)	9.0	13.0	7.9
Calculated monomeric Mr from sequence (kDa	7.8	12.2	4.4

Software employed

Primary data reduction	Sangler 2.1.39
Guinier Analysis	AutoGuinier (ATSAS 3.1.3)
Zero-concentration Extrapolation	MOLASS 1.0.10
Data processing	PRIMUS
