# Supplementary material for: Structure of human DPPA3 bound to the UHRF1 PHD finger reveals its functional and structural differences from mouse DPPA3
Source: Commun Biol. 2024 Jun 19;7:746. doi: 10.1038/s42003-024-06434-9 (PMC11187062; doi:10.1038/s42003-024-06434-9)
Supplement: Supplementary file 3 — Description of additional supplementary files [file 42003_2024_6434_MOESM3_ESM.docx]

**Description of Additional Supplementary Files**

File name: Supplementary Data 1

Description: Source data for Figure 1c and Supplementary Figure 4a.

File name: Supplementary Data 2

Description: Source data for Figure 3a.

File name: Supplementary Data 3

Description: Source data for Figure 3b, 3c, and Supplementary Figure 3a-c.

File name: Supplementary Data 4

Description: Source data for Figure 4b, Figure 5, and Supplementary Figure 4b.
