# Supplementary material for: Structure of human DPPA3 bound to the UHRF1 PHD finger reveals its functional and structural differences from mouse DPPA3
Source: Commun Biol. 2024 Jun 19;7:746. doi: 10.1038/s42003-024-06434-9 (PMC11187062; doi:10.1038/s42003-024-06434-9)
Supplement: Supplementary file 7 — Supplementary data 4 [file 42003_2024_6434_MOESM7_ESM.pdf]

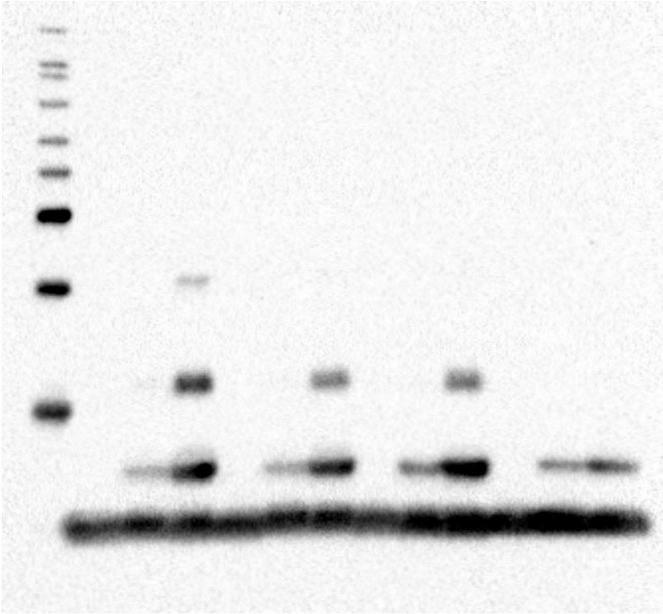

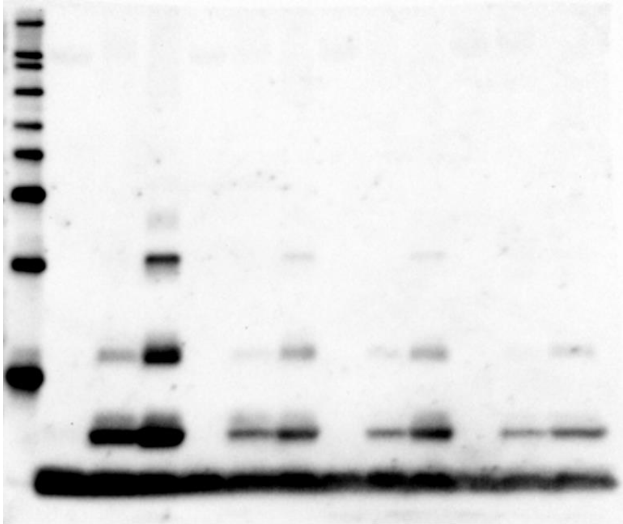

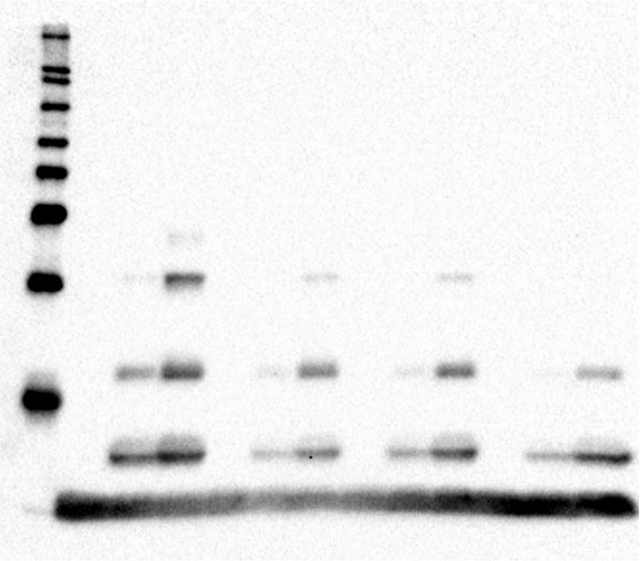

Uncropped blot images related to the top panel of Figure 4b.

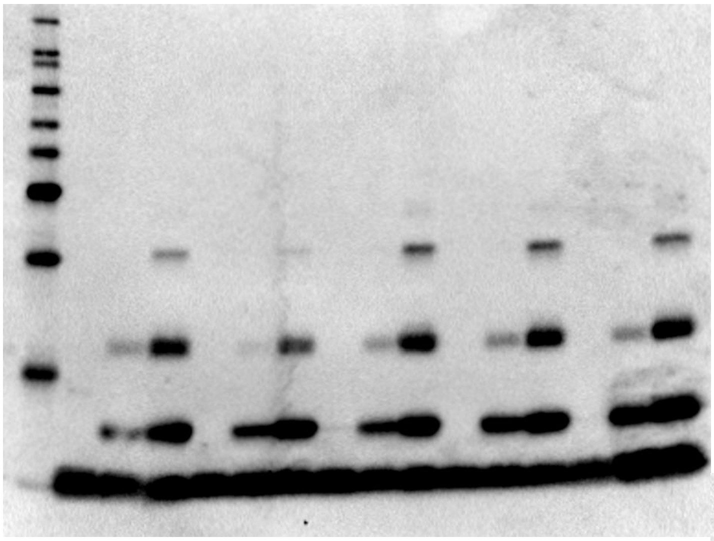

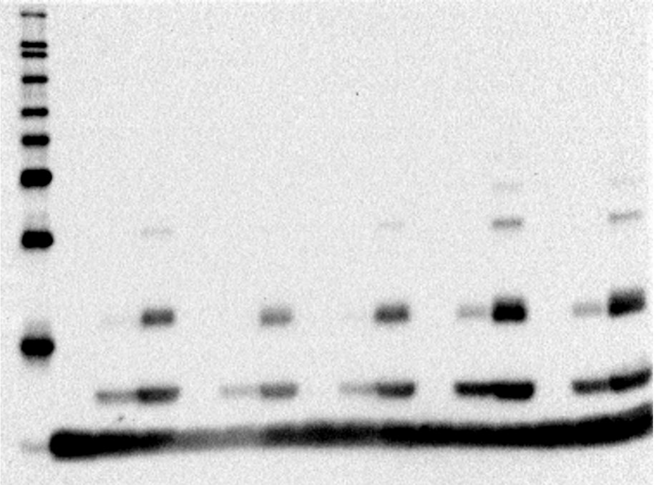

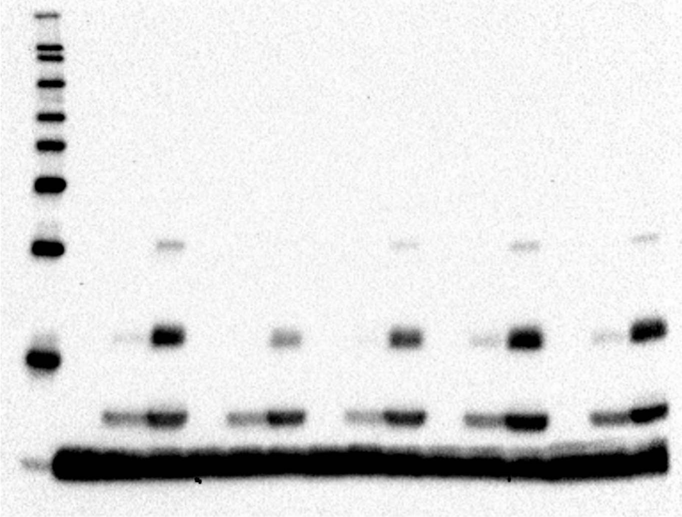

Uncropped blot images related to the bottom panel of Figure 4b.

c

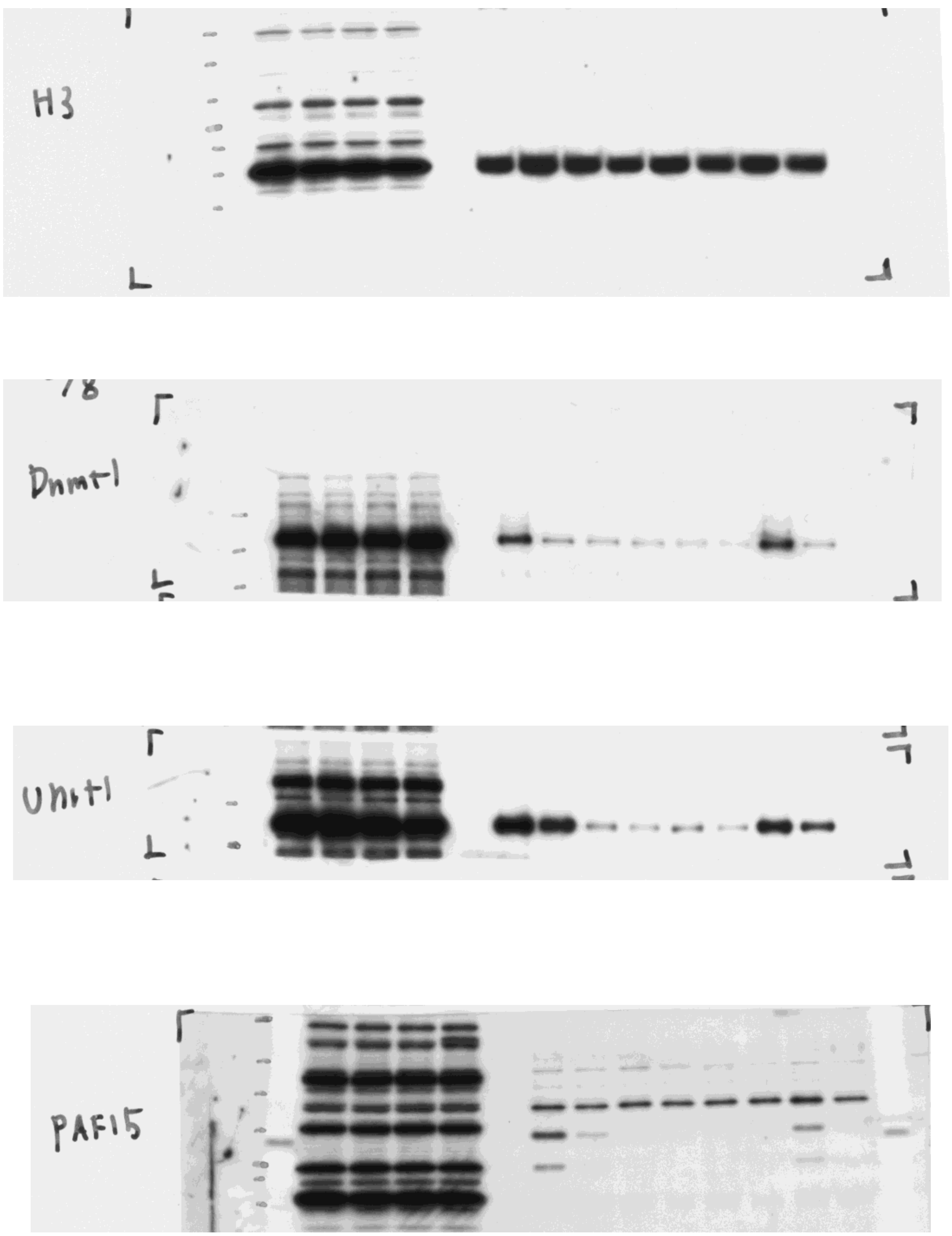

Uncropped blot images related to Figure 5b.

d

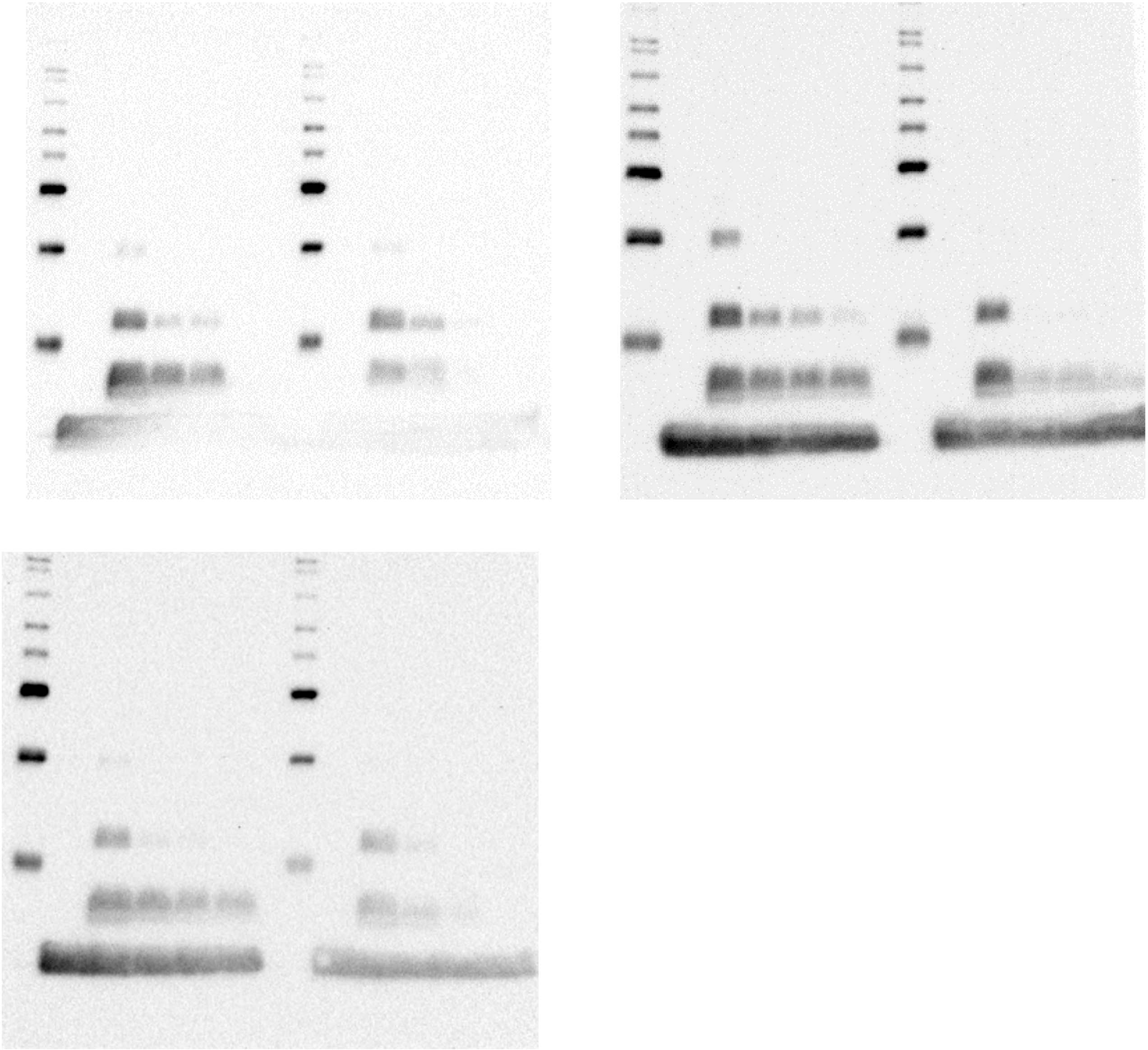

Uncropped blot images related to Supplementary Figure 4b.
